# Draft Genome Sequences of Non-H_2_S-Producing Strains of Salmonella enterica Serovars Infantis, Enteritidis, Berta, and Kiambu in Japan

**DOI:** 10.1128/MRA.00335-20

**Published:** 2020-07-23

**Authors:** Hiroshi Asakura, Junko Sakata, Shiori Yamamoto, Shizunobu Igimi

**Affiliations:** a Division of Biomedical Food Research, National Institute of Health Sciences, Kanagawa, Japan; b Department of Bacteriology, Osaka Institute of Public Health, Osaka, Japan; c Department of Microbiology, Tokyo University of Agriculture, Tokyo, Japan; Indiana University, Bloomington

## Abstract

We report the draft genome sequences of six strains of Salmonella enterica serovars Berta, Enteritidis, Infantis, and Kiambu, isolated from humans or chicken meats in Osaka, Japan, that were negative for hydrogen sulfide production. Their genome sizes ranged from 4,460,389 to 4,933,483 bp, with 3 to 9 rRNAs and 64 to 73 tRNAs and with coverages of 95× to 159×.

## ANNOUNCEMENT

Salmonella enterica subsp. *enterica* is one of the leading causes of foodborne gastrointestinal illnesses, some of which develop into invasive salmonellosis (1). According to a Japanese governmental report, a total of 640 individuals suffered from foodborne salmonellosis, representing approximately 3.7% of overall annual foodborne illness patients (17,282 individuals), in 2018 in Japan (2). Epidemiology-based source attribution studies have provided evidence that eggs and chicken meats are some of the main sources of human salmonellosis worldwide (3). Traditionally, hydrogen sulfide (H_2_S) production has been used as one of the biochemical hallmarks to selectively isolate this pathogen through bacteriological culture procedures (4–6). However, non-H_2_S-producing *Salmonella* spp. have been increasingly detected in human and food specimens (7, 8).

To characterize the genomic traits of non-H_2_S-producing *Salmonella* spp., the draft genome sequences of six strains, S. enterica serovar Berta SB12A043, S. enterica serovar Enteritidis SE03A200, S. enterica serovar Infantis SI19A061, SI23A178, and SI12A186, and S. enterica serovar Kiambu SK20A094, which originated from feces from human patients with gastroenteritis or from food specimens (chicken meats), were examined. For bacterial isolation from human feces, samples were preenriched in buffered peptone water (BPW) (Oxoid, UK) at 37°C for 24 h, followed by plating onto *Salmonella*-*Shigella* (SS) agar (Eiken Kagaku, Tokyo, Japan). The suspected white colonies were confirmed to be *Salmonella* spp. by their antigenic characterization based on the Kauffmann-White scheme using *Salmonella* antiserum Seiken (Denka, Tokyo, Japan). Bacterial isolation from food specimens was performed as follows: 25 g of the samples was preenriched in 225 ml of BPW at 37°C for 24 h, followed by selective enrichment using Rappaport-Vassiliadis broth (Oxoid) at 37°C for 22 h. An aliquot of the culture was then spread onto brilliant green sulfa agar (Becton, Dickinson and Company, Franklin Lakes, NJ, USA) and incubated at 37°C for 24 h. The suspected colonies, appearing red to pink-white surrounded by a red zone, were subjected to serotyping for bacterial identification and classification, as described above. Finally, the strains obtained were genetically confirmed to be *Salmonella* spp. by PCR assays, as described (9).

After cultivation in BPW for 18 h at 37°C with shaking, 1 ml of the culture was used to extract genomic DNA from the six strains by using the Maxwell RSC blood DNA kit (Promega, Madison, MA, USA) with minor modifications. In brief, the bacterial pellet obtained by centrifugation (14,500 × *g* for 5 min) of 1 ml of BPW culture was resuspended in 400 μl of homogenization solution (Promega), followed by homogenization with a ZircoPrep minikit (Nippon Genetics, Tokyo, Japan) on a Digital Disruptor Genie (Scientific Industries, Bohemia, NY, USA) for 5 min at 2,850 rpm. After centrifugation at 10,000 × *g* for 5 min, 100 μl of the sample lysates and 300 μl of lysis buffer were added to the deep-well processing plate supplied in the kit, and DNA extraction was performed in a Maxwell RSC instrument (Promega) with default settings. The concentration and purity of the isolated DNA were checked with a TapeStation 4150 system (Agilent Technology, Santa Clara, CA, USA), and the exact concentration was determined using the Qubit double-stranded DNA (dsDNA) high-sensitivity (HS) assay kit, as recommended by the manufacturer (Thermo Fisher Scientific, Waltham, MA, USA). Each 1-μg sample of genomic DNA was used to construct a library by using the Ion Xpress Plus fragment library kit (Thermo Fisher Scientific). The sequencing reaction was performed in an Ion GeneStudio S5 sequencer using the Ion 530 kit in combination with the Ion 530 chip (Thermo Fisher Scientific). Raw reads were trimmed and *de novo* assembled using CLC Genomics Workbench v20 (Qiagen, Hilden, Germany). The parameters for trimming were as follows: ambiguous limit, 2; quality limit, 0.05; number of 5′-terminal nucleotides, 20; and number of 3′-terminal nucleotides, 5. The parameters for the *de novo* assembly were as follows: mapping mode, create simple contig sequences (fast); bubble size, 50; word size, 20; minimum contig length, 1,000 bp; and perform scaffolding, no.

### Data availability.

The draft genomes of all six strains have been deposited in DDBJ/EMBL/GenBank under the accession numbers provided in Table 1.

**TABLE 1:** Strain and genome information for the current study

Strain name	Serovar	Source	Yr	No. of reads	No. of contigs	Size (bp)	*N_50_* (bp)	GC content (%)	Coverage (×)	DDBJ accession no.	SRA accession no.
SB12A043	Berta	Human feces	2000	318,248	79	4,791,818	105,935	52.3	159.0	BLQG010000000	DRR190999
SE03A200	Enteritidis	Human feces	1991	198,280	91	4,831,261	91,788	52.0	95.1	BLQH01000000	DRR191000
SI19A061	Infantis	Chicken meat	2007	226,712	99	4,878,412	96,185	52.2	119.1	BLQI01000000	DRR191001
SI23A178	Infantis	Chicken meat	2011	265,963	100	4,933,483	75,389	52.1	143.8	BLQJ01000000	DRR191002
SI23A186	Infantis	Chicken meat	2011	233,400	97	4,813,141	98,441	52.1	114.5	BLQK01000000	DRR191003
SK20A094	Kiambu	Human feces	2008	227,083	69	4,460,389	121,564	52.2	114.7	BLQL01000000	DRR191004
